# Mushrooms: a food-based solution to vitamin D deficiency to include in dietary guidelines

**DOI:** 10.3389/fnut.2024.1384273

**Published:** 2024-04-10

**Authors:** Carlene Starck, Tim Cassettari, Jutta Wright, Peter Petocz, Emma Beckett, Flavia Fayet-Moore

**Affiliations:** ^1^FOODiQ Global, Sydney, NSW, Australia; ^2^Macquarie University, Sydney, NSW, Australia; ^3^School of Health Sciences, The University of New South Wales, Kensington, NSW, Australia; ^4^School of Environmental and Life Sciences, The University of Newcastle, Callaghan, NSW, Australia

**Keywords:** vitamin D deficiency, vitamin D intakes, mushrooms, dietary guidelines, recommended intake

## Abstract

Vitamin D deficiency and insufficiency is a public health issue, with low dietary vitamin D intakes a contributing factor. Rates of vitamin D deficiency are 31% in Australia, and up to 72% in some regions globally. While supplementation is often prescribed as an alternative to additional sun exposure, complementary approaches including food-based solutions are needed. Yet, food-centric dietary guidelines are not always adequate for meeting vitamin D needs. Edible mushrooms such as *Agaricus bisporus* can produce over 100% of vitamin D recommendations (10 μg/day, Institute of Medicine) per 75 g serve (18 μg) on exposure to UV-light, with the vitamin D_2_ produced showing good stability during cooking and processing. However, mushrooms are overlooked as a vitamin D source in dietary guidelines. Our dietary modelling shows that four serves/week of UV-exposed button mushrooms can support most Australian adults in meeting vitamin D recommendations, and UV-exposed mushrooms have been found to increase vitamin D status in deficient individuals. While recent evidence suggests some differences between vitamin D_2_ and vitamin D_3_ in physiological activities, vitamin D_2_ from mushrooms can be part of a larger solution to increasing dietary vitamin D intakes, as well as an important focus for public health policy. Mushrooms exposed to UV represent an important tool in the strategic toolkit for addressing vitamin D deficiency in Australia and globally. Health authorities lead the recognition and promotion of mushrooms as a natural, vegan, safe, and sustainable vitamin D food source.

## Introduction: vitamin D deficiency is a global problem

Vitamin D deficiency is a global health concern with significant implications for population health. Systematic reviews (1–6) indicate that rates of vitamin D deficiency, when defined as blood level <50 nmol/L (7, 8), are as high as 47.9% globally (range 19 to 72%) (Table 1) (1). In Australia, approximately three-quarters of the adult population have suboptimal vitamin D status; the rate of vitamin D deficiency is 31% (1) and insufficiency (50 to 75 nmol/L) a further 43% (16). Some population groups are more vulnerable, with deficiency rates up to 94% in residential care-elderly (17, 18). Along with a well-established role in bone health (19), associations have been made between vitamin D inadequacy and increased susceptibility to infectious diseases (including COVID-19), muscle weakness, multiple sclerosis, diabetes, hypertension, metabolic syndrome, cancers, autoimmune diseases, cardiovascular disease (1), and gestational diabetes (20). While these associations are largely observational, the evidence is clear: the “sunshine” vitamin requires a metaphorical light to be shone upon it, and on a global scale.

**Table 1:** Rates of vitamin D deficiency and mean vitamin D intakes in Australia and World Health Organization global regions.

Country/region^c^	Rate of vitamin D deficiency^a^	Mean vitamin D intake (μg/day)^b^	Additional references
Australia	31%	1.84 to 3.25	(8)
Africas	19%	1 to 9.6	(9)
Eastern Mediterranean	72%	1 to 4	(10)
Europe	53%	2 to 4	(10–13)
Americas	30%	3.5 to 6	(14, 15)
South-East Asia	57%	1.5 to 5.5	(11)
Western Pacific	44%	1.84 to 7.6	(8, 11)

a World Health Organisation (WHO) regions (1), with Australia considered separately.

b Defined as <50 nmol/L (7, 8). Rates of deficiency sourced from Cui 2023 meta-analysis (1).

c Intake data sourced from the references listed in the final column of this table.

### Food-based solutions can support vitamin D intake and status

Current vitamin D guidelines in Australia suggest that sunlight is a key source of vitamin D; where sunlight exposure is limited, supplements are the recommended alternative, with diet considered a poor source (8, 18, 21–23). Conversely, increased vitamin D intake from foods has consistently shown the ability to improve vitamin D status in those who have sub-optimal status (24). Global vitamin D intakes are lower than the estimated average requirement (EAR) for vitamin D of 10 μg/day set by the Institute of Medicine (IOM) (25) (Table 1) and recommended dietary patterns provided by dietary guidelines are insufficient for vitamin D (21, 26).

Australian intakes of vitamin D are among the lowest in the world at 1.84 to 3.25 μg/day (8). While the potential for daily sunlight exposure is high in countries such as Australia, levels of exposure are insufficient to maintain vitamin D adequacy, with many factors suggested to play a role, including indoor lifestyles, skin color, and skin cancer risk (27). Vitamin D from sunlight exposure also varies according to season, with a 1.7 fold higher global vitamin D deficiency rate between winter/spring compared to summer/autumn, and higher rates in people living in areas of high latitude (1). An increased focus on addressing and improving vitamin D intake is needed, and the strategies and policies suggested include vitamin D supplementation, an increased intake of foods naturally high in vitamin D, traditional food fortification with vitamin D, and biofortification of vitamin D food sources (8).

Vitamin D supplementation is important for vulnerable groups such as the elderly, and those following a diet that restricts animal foods (1, 2, 17, 28). In Australia, vitamin D supplementation ranges from 0.6 to 17% depending on demographic group, with the highest rates among elderly women (29). Low adherence within the general population and reduced accessibility by those of low socioeconomic status are significant limitations for widespread application to address vitamin D deficiency (1, 2, 17, 24). Supplements are predominantly vitamin D_3_, sourced from sheep wool, which is incompatible with a vegan diet (30). Vegan vitamin D_3_ supplements from microalgae such as *Nannochloropsis oceanica* can be produced when they are irradiated with UV (31). Similarly, mushrooms can produce vitamin D_2_ when irradiated with UV light (32) and vegan vitamin D_2_ supplements made from mushrooms and other fungi are available. While 7-dehydrocholesterol is converted to vitamin D_3_ in animals, ergosterol (pro-vitamin D_2_), which is found in mushrooms, is converted to pre-vitamin D_2_ under UV-light, with heat required for full conversion to ergocalciferol, vitamin D_2_ (32).

The highest non-fortified and natural food sources of vitamin D are predominantly animal-based, such as salmon (5.4 μg/100 g) and eggs (5.9 μg/100 g) (33). Animal-based solutions do not align with plant-based movements or vegan diets. A recent simulation study suggested that the achievement of adequate vitamin D intake based primarily on animal food sources is not possible within carbon emission limits (34).

Food fortification with vitamin D has been shown to be both efficacious and cost-effective (24). In Finland, voluntary vitamin D fortification of milks and margarines/fat spreads was associated with an increase in mean vitamin D status from 47.6 nmol/L to 65.4 nmol/L over 11 years (24). In the US, fortified milk and milk products provide the greatest contribution to dietary vitamin D intake (43.7%) (35) and UV-exposed mushroom powder is approved by the FDA as a vitamin D_2_ source for addition to some foods (36). Fortified margarine is a major dietary source of vitamin D in Australia (37). Australian dietary modelling showed that fortification of milk and breakfast cereals with vitamin D (1 μg/100 mL and 3.5 μg/100 g respectively) would increase average vitamin D intake from 3.6 μg/day to 6.3 μg/day, although remaining below IOM targets (38).

Biofortification describes the natural vitamin D enrichment of whole foods including meats, eggs, and fish (via increased vitamin D provision to livestock), as well as UV-exposed mushrooms and yeast (39, 40). For example, the vitamin D_3_ content of eggs can be increased by the addition of vitamin D_3_ to the feed of hens (40). In the US, both vitamin D-enriched and sunlight-exposed mushrooms are readily available for purchase, containing 100% of the IOM EAR per 85 g serving (25, 41). In Australia, vitamin D-enriched mushrooms are produced via exposure to UV-lamp pulses (42).

There is some uncertainty around the potential for excess intake of vitamin D, given the increase in nutrient availability from fortification and high-dose vitamin D supplements (43), although mean intake estimates are less than 10% of the IOM upper level of intake (UL) (1). An updated assessment of vitamin D intake from all sources is warranted.

### Mushrooms produce the recommended intake of vitamin D (as D_2_) per serve

The vitamin D content of UV-exposed mushrooms varies according to mushroom type, the amount of UV-exposure, the surface area exposed (whole or sliced), light intensity, and length of exposure (28). Australian UV-exposed white button mushrooms can provide over 100% of vitamin D requirements in a single serve (Australian Guide to Healthy Eating, AGHE) (28, 33). In Germany, 100 g of sliced *A. bisporus* mushrooms exposed to midday, mid-summer sunlight produced 17.5 μg vitamin D_2_ after 15 min and 32.5 μg after 60 min (44), 175 and 325% of the IOM EAR, respectively (25). UV-lamp pulses (1–2 s) after harvest produced 24 μg/100 g (240% EAR) (33). The vitamin D content of UV-exposed *A. bisporus* mushrooms is notably higher than other dietary vitamin D sources including both oily fish (5.7 μg) and eggs (7.1 μg) per serve (33, 45). Caution is warranted as there are reports of vitamin D concentrations up to 320 μg/100 g (3,200% EAR) with pulsed UV exposure, above the IOM UL (28). Commercial production of vitamin D enhanced mushrooms requires standardization and testing to stay within the upper limit.

The Australian Food Composition Database reports that even non-UV exposed white button mushrooms can be a source of vitamin D in Australia, providing 16% of the IOM EAR per serve (25, 33). This is in contrast to levels stated in food composition databases in the US (46) and New Zealand (47), at 0.02 μg and 0 μg vitamin D/100 g, respectively. As mushrooms do not naturally contain vitamin D without UV exposure, it is likely that the mushrooms analyzed at point of sale in Australia had incidental UV exposure post-harvest.

### Mushrooms are a feasible and sustainable food-based source of vitamin D

Post synthesis, the vitamin D_2_ content of UV-exposed *A. bisporus* mushrooms remains largely stable for around 7 to 10 days when refrigerated (28). The retention of vitamin D_2_ during cooking ranges from 62 to 88%, depending on cooking method, with the highest retention in mushrooms pan-fried without oil (48). UV-exposed mushrooms are therefore a feasible, food-based source of vitamin D, consumed raw or cooked. It is unknown how cooking affects the bio-accessibility of vitamin D from mushrooms in humans; this deserves further investigation as the bio-accessibility of some nutrients is enhanced following cooking.

UV-exposed mushrooms as a vitamin D source support sustainability efforts. The notably low environmental impact of mushrooms is largely due to their role in circular agriculture, supporting the growth, maintenance, and remediation of the surrounding environment (49, 50). In circular agriculture, outputs from plant and animal farm waste are used as inputs in mushroom growing, and spent mushroom waste is then used to produce high-quality compost, animal feed, biofuel, and for bioremediation (49, 50).

### Mushrooms are not considered as a vitamin D source in dietary guidelines

Recommended intakes for vitamin D vary worldwide; while the IOM recommends an EAR of 10 μg/day, and RDA up to 20 μg/day for adults over 70 years (25), Australian recommendations are based around adequate intake (AI), ranging from 5–15 μg/day depending on age group, with the highest requirements for those aged 65 years and older (37). Despite these recommendations, food-based dietary guidelines often lack provision for vitamin D; both the Australian Dietary Guidelines (ADG) and the Dietary Guidelines for Americans (DGA) (26) fail to provide adequate vitamin D (21, 26), indicating that meeting vitamin D needs is difficult and current eating patterns require additional support. Further, vitamin D does not feature as a characteristic essential nutrient in any of the ADG core food groups.

Similarly, the role of UV-exposed mushrooms in the provision of vitamin D is not recognized within dietary guidelines worldwide. The AGHE considers mushrooms within the “vegetables and legumes” subcategory of “other vegetables,” alongside salad vegetables such as tomatoes and cucumber (45). Other vegetable subcategories include dark green or cruciferous vegetables, root vegetables, and legumes/beans (45). Mushrooms are also classified as “other vegetable” in the DGA (51). Neither the American (52) nor Australian (21) dietary modelling approaches that underpin guideline development considered the use of UV-exposed mushrooms as a source of vitamin D. The Australian modelling included mushrooms at around 2% of total vegetable consumption for adult diets (less than 50 g per week), consistent with recent sales data (53).

### Dietary modelling supports a role for UV-mushrooms as a key vitamin D source

In 2021, two dietary modelling papers from the USA (54, 55) showed that the daily addition of an 84 gram serve of UV-exposed *A. bisporus* mushrooms (containing 5 μg of vitamin D) dramatically improved vitamin D intakes (by 67 to 91%, depending on the baseline diet) and decreased vitamin D inadequacy in the usual US adult diet from 94.9 to 63.6%, with minimal impacts on energy and sodium levels.

To determine Australian-based dietary outcomes of UV-exposed mushroom addition, we modelled the effect of removing mushrooms from the “other vegetables” sub-category of the “vegetables and legumes” core food group and creating a fifth “mushrooms only” sub-category of vegetables and legumes. Mushrooms were then added to the diet as this fifth sub-category with increasing numbers of serves. This was carried out for three adult demographic groups (women aged 19–30 years, men aged 51–70 years, and women aged >70 years) and two diets (omnivore and ovo-lacto vegetarian). Methodological detail is provided in Supplementary Figure S1.

**Figure 1:**
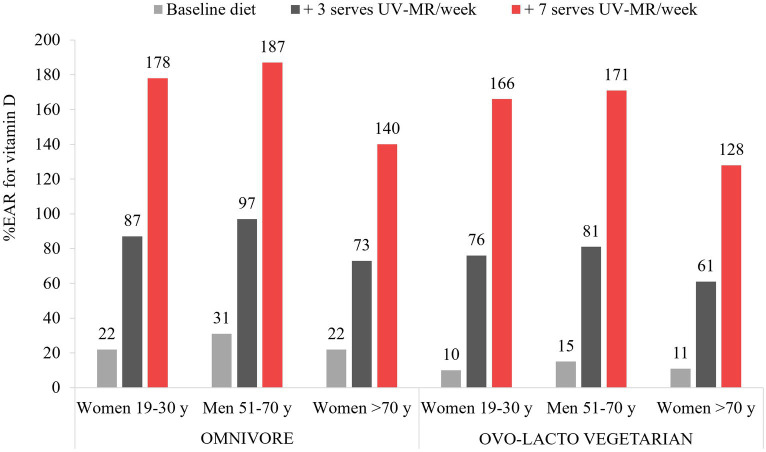
Vitamin D intakes as a percentage of the Institute of Medicine Recommended Daily Allowance for vitamin D for each demographic group, as a result of dietary modelling focused on mushrooms as a separate subcategory of vegetables within Foundation Diets from the Australian Dietary Guidelines. One serve is equivalent to 75 g.

The modelling showed notable increases in vitamin D intakes, as well as several additional micronutrients, for all demographic groups and both diet models (omnivore and ovo-lacto vegetarian). While all baseline diets were inadequate for vitamin D, ranging from 10 to 31% of the IOM RDA across demographic groups, the addition of one serve/day (75 g) of UV-exposed mushrooms enabled all demographic groups to exceed their recommended dietary vitamin D intake by 28 to 87% (Figure 1). Recommendations were achieved at a minimum of 4 serves per week for adults up to the age of 70 years; over this age, 5 and 6 serves per week for the omnivore and ovo-lacto vegetarian diet, respectively, were required (data not shown). Beneficial effects on additional micronutrients included an 18.6 to 34.2% increase in selenium across all demographic groups and both diets (Supplementary Table S1). While there were small decreases in some nutrients in substitution models, such as riboflavin and vitamin B12, NRVs were still met. There was a negligible impact on energy intakes in all diets, even when 7 serves of UV-exposed mushrooms were added to current recommendations per week.

The findings show that UV-exposed mushrooms have the potential to make a meaningful contribution to vitamin D intakes of Australian adults, allowing an individual to meet their vitamin D needs if consumed daily. However, the modelled intake (525 g/week) is notably higher than current intakes, both in Australia (50 g/week) (53) and globally (100 g/week) (56). Education, policy, and programs on the benefits of UV-exposed mushrooms alongside their production across major suppliers may offer an effective solution to addressing low vitamin D intakes and inadequacy. Although the consumption of UV-exposed mushrooms may not, in practice, consistently align with the amounts necessary to fully provide for recommended vitamin D intakes, UV-exposed mushrooms can play an important role as part of the solution to low vitamin D intakes, supporting other dietary sources of vitamin D, as well as supplementation where necessary. This is particularly important for those consuming vegetarian and vegan diets, where there is a low intake of animal-based vitamin D. Future dietary modelling in dietary guidelines needs to consider UV-exposed mushrooms as a source of vitamin D.

The modelling approach presented here was focused on adult demographic groups only; however, UV-exposed mushrooms may also represent an additional vitamin D source for children. While mushrooms contain insoluble fibres such as chitin, and excessive intakes of fibre may be associated with gastrointestinal discomfort in this population (57), the prevalence of vitamin D deficiency in Australian children is lower than that in adults (58), indicating that a reduced consumption of UV-exposed mushrooms may support adequate intakes. Further research to determine consumption levels in children and effect on vitamin D status is warranted.

### Is D_2_ from mushrooms a substitute for D_3_?

The efficacy of vitamin D_2_ compared to D_3_ for increasing vitamin D status (25-hydroxyvitamin D, 25 (OH)D) is yet to be fully understood. While meta-analyses have shown that vitamin D_3_ is more effective than vitamin D_2_ in increasing total vitamin D status, these relationships appear to be modified by both BMI and baseline vitamin D status (59). A 2023 systematic literature review identified that vitamin D_2_ from UV-exposed mushrooms (from 8.8 μg/day) consistently increased serum levels of vitamin D_2_ compared to placebo (60). However, there was no change in total vitamin D levels in most studies, possibly explained by the concomitant decrease in vitamin D_3_ levels in 50% of studies. This may reflect a tight regulation of total vitamin D levels (61). In one trial, there was a decrease in total vitamin D that was greater in subjects with higher vitamin D at baseline (62). A parallel RCT not included in the 2023 review found that consumption of mushrooms containing D_2_ was as effective at increasing and maintaining total serum vitamin D levels as both supplemental vitamin D_2_ and D_3_ (all 50 μg/day) (63). In this study, baseline vitamin D was bordering deficiency. A 2024 systematic review with meta-analysis found no effect of mushroom vitamin D_2_ on serum vitamin D status, although significance was borderline (*p* = 0.06) and a statistically and clinically significant increase was seen in sub-analyses of the lowest (42 nmol/L) vs. highest (>75 nmol/L) baseline vitamin D status (64). Similarly, vitamin D_2_ supplementation-induced decreases in vitamin D_3_ appear to be highest in those with highest baseline vitamin D (64). The relative effectiveness of vitamin D_2_ and D_3_ for increasing vitamin D status also appears to depend on level and frequency of dose, with larger differences between D_2_ and D_3_ in bolus compared to daily dosing protocols (65, 66). Despite these differences, pharmacologic doses of vitamin D_2_ have shown the ability to maintain serum vitamin D above 50 nmol/L in clinical settings of vitamin D deficiency (67, 68). Together, the evidence suggests that beneficial increases in vitamin D status can occur with vitamin D_2_ from mushrooms in those with deficient or insufficient vitamin D status.

A key aspect of vitamin D biology that requires further understanding is the physiological role of D_2_ vs. D_3_. While sharing a similar structure, differences in the half-life of the hydroxylated forms of vitamin D_2_ and D_3_ have been identified (D_2_ is shorter), at least in some populations (69). There may also be differences in the rate of hydroxylation, affinity for the vitamin D binding protein, and binding to the vitamin D receptor (61). A recent analysis of the blood transcriptome following D_2_ vs. D_3_ supplementation showed that, despite there being overlap in gene expression changes, some were specific to one form of the vitamin vs. the other (70).

Together, the findings suggest that while vitamin D_2_ is not a direct substitute for vitamin D_3_, vitamin D enhanced mushrooms can increase vitamin D status among those who are deficient or insufficient, those with low potential for UV exposure, and those with limited intake of animal sources of vitamin D. A more comprehensive analysis of the biological effects of the two forms of vitamin D in humans is needed.

### Call to action: promoting UV-exposed mushrooms as part of the solution to vitamin D deficiency

UV-exposed mushrooms show potential to be a meaningful, whole-food, and vegan source of vitamin D. Current dietary guidelines are increasingly focused on environmental sustainability and “plant-based” diets; while possessing numerous benefits, such guidelines may inadvertently increase vitamin D deficiency and suboptimal intakes of other micronutrients, such as selenium.

There is an opportunity for health professionals, stakeholders, and policy makers to provide greater guidance on maximizing diet as a source of vitamin D, by UV-exposing mushrooms and increasing their focus in dietary guidelines worldwide. Moving mushrooms into a new sub-category of the vegetables core food group, as we have modelled, could facilitate an increase in mushroom intake. Practical guidance in using and cooking mushrooms as a major source of vitamin D can be provided, such as purchasing UV-exposed mushrooms or putting mushrooms in the sun prior to eating (15 min in the sun between 10 am and 3 pm, and store in fridge for up to 8 days (28)).

The production of UV-exposed mushrooms is limited and comes at a cost to farmers and consumers, creating a barrier for its inclusion. Government action in supporting the universal UV-exposure of mushrooms, similarly to efforts in the universal iodization of salt (71), could enhance efforts to address vitamin D deficiency globally. UV-exposed mushrooms can contribute to addressing vitamin D inadequacies in a sustainable, whole-food fashion, warranting their consideration as a key and substantial dietary source of vitamin D.

## Data availability statement

The original contributions presented in the study are included in the article/Supplementary material, further inquiries can be directed to the corresponding author.

## Ethics statement

Ethical approval was not required for the study involving humans in accordance with the local legislation and institutional requirements. Written informed consent to participate in this study was not required from the participants or the participants’ legal guardians/next of kin in accordance with the national legislation and the institutional requirements.

## Author contributions

CS: Conceptualization, Data curation, Formal analysis, Investigation, Methodology, Writing – original draft, Writing – review & editing. TC: Conceptualization, Methodology, Writing – review & editing. JW: Writing – review & editing. PP: Methodology, Validation, Writing – review & editing. EB: Writing – review & editing. FF-M: Funding acquisition, Methodology, Supervision, Writing – review & editing.
