# Supplementary material for: Mushrooms: a food-based solution to vitamin D deficiency to include in dietary guidelines
Source: Front Nutr. 2024 Apr 10;11:1384273. doi: 10.3389/fnut.2024.1384273 (PMC11039838; doi:10.3389/fnut.2024.1384273)
Supplement: Supplementary file 1 [file Data_Sheet_1.pdf]

Supplementary Material

1. Supplementary Methodology

Methodology used for dietary modelling of mushrooms as a separate subcategory of vegetables.

The modelling approach was based on that used for the 2013 revision of the ADG (1) and was performed at the level of Foundation Diets (designed to meet the energy needs of the smallest and most inactive members of a demographic group), for two dietary patterns (omnivore and ovo-lacto vegetarian) and for three adult demographic groups (women 19-30 years; men 51-70 years; women over 70 years). The selected dietary models were based on difference in potential food sources of vitamin D and potential for vitamin D inadequacy (2). Vitamin D requirements were based on the Institute of Medicine (IOM) Recommended Daily Allowance (RDA) of 15 µg/day for all demographic groups except adults over 70 years, for which the RDA is 20 µg/day. The total nutritional composition of each modelled Foundation (baseline) diet for each demographic group per week and per day was calculated, as well as the ability of each diet to meet NRVs. For all nutrients, AI or RDI (IOM RDA for vitamin D) were used in place of EAR to represent the nutrient requirements of most individuals within each demographic group (1)). The nutritional composition of UV-exposed mushrooms was based on a ratio of 50% raw and 50% cooked (3).

The modelling was carried out over three steps (shown in **Supplementary Figure 1**):

Step 1. A modelling database was first created using the nutritional composition of each Foundation Diet for each demographic group provided within the Australian Dietary Guidelines (ADG) modelling document (1) and supported by Australian Food Composition Database (AFCD) data (3).

Step 2. Two alternatives to the original "other vegetables" sub-category were created: "other vegetables - no mushrooms" which included the original sub-category group without mushrooms; and, "UV-exposed mushrooms", containing only UV-exposed mushrooms (3). The composition of the new mushrooms sub-category was an average of raw and cooked (no fat included) UV-exposed mushrooms. The "other vegetables – no mushrooms" subcategory was created by subtracting the calculated nutritional composition of mushrooms (non-UV exposed) from the original "other vegetables" subcategory, followed by normalising the relative percentage contribution of all remaining vegetables to equal 100%. One serve was 75 grams, as specified by the ADG (4).

Step 3. The modelling of mushrooms as a separate sub-category of vegetables was conducted by subtracting the nutritional composition of "other vegetables – mushrooms" from the total nutritional composition of each original Foundation Diet, for each demographic group, and adding back varying serve numbers of each of "other vegetables – no mushrooms" or the UV-exposed mushrooms only sub-category. This was carried out for a weekly diet in the first instance, within division by 7 to achieve a daily diet. The number of recommended serves (each being 75 g) of "other vegetables" within omnivore and ovo-lacto vegetarian dietary patterns per week is 14 and 7, respectively (1).

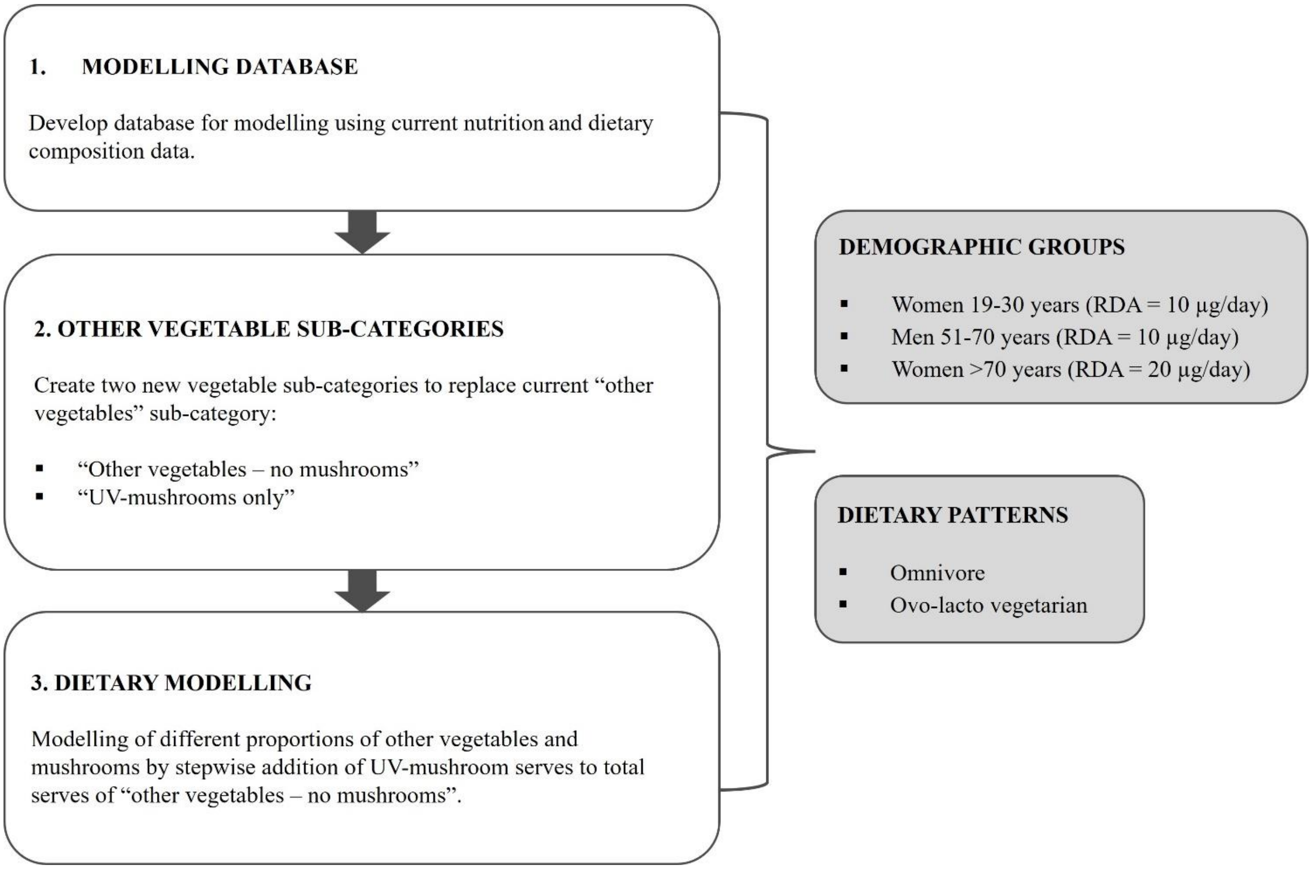

Supplementary Figure 1. Summary of modelling approach used to estimate the impact of UV-exposed mushrooms on vitamin D intakes, when mushrooms are included as a separate sub-category of the vegetables core food group.

2. Supplementary Tables

Supplementary Table 1. Full results of the mushroom-centred dietary modelling (one serve per day) for each demographic group and for both omnivore and ovo-lacto vegetarian diets, showing the percentage change in each nutrient compared to the *Foundation Diet* produced by ADG dietary modelling.

Dietary nutrient	Women 19-30 years			Men 51-70 years			Women >70 years		
	**FDN diet**	**+ UV-MR**	**% Δ**	**FDN diet**	**+ UV-MR**	**% Δ**	**FDN diet**	**+ UV-MR**	**% Δ**
**Omnivore diet**									
Macronutrients									
Energy (kJ)	7384	7461.9	1.05	8286.5	8364	0.94	6586.6	6665.1	1.19
Protein (g)	101.2	103.3	2.08	104.9	107.1	2.10	94.1	96.3	2.34
Carbohydrate (g)	206.3	207.9	0.78	214.8	216.3	0.70	169.5	170.9	0.83
Sugars (g)	91.1	91.4	0.33	91.9	92.2	0.33	108.2	108.4	0.18
Dietary fibre (g)	34	35.5	4.41	34.4	35.9	4.36	26	27.5	5.77
Total fat (g)	52.4	52.8	0.76	70.9	71.3	0.56	52.6	53	0.76
SFA (g)	17.8	17.9	0.56	21.9	22	0.46	19.1	19.2	0.52
Vitamins									
Thiamin (mg)	1.6	1.6	0.00	1.6	1.7	6.25	1	1.1	**10.00**
Riboflavin (mg)	2.5	2.5	0.00	2.5	2.5	0.00	2.9	2.9	0.00
Niacin eq.(mg)	51.5	55	6.80	51.8	55.4	6.95	45.8	49.6	8.30
Vitamin B6 (mg)	1.7	1.8	5.88	1.8	1.8	0.00	1.7	1.7	0.00
Folate (DFE) (µg)	496.3	515.2	3.81	494.7	513.5	3.80	512.9	531.8	3.68
Vitamin B12 (µg)	5.9	5.9	0.00	6.2	6.2	0.00	8.1	8.1	0.00
Vitamin A (RE) (µg)	1435.6	1440.2	0.32	1575.8	1578.8	0.19	1366.6	1368.2	0.12
Vitamin C (mg)	125.3	128.3	2.39	129.2	131.4	1.70	122.4	123.4	0.82
Vitamin D (µg)	3.3	26.6	**706.06**	4.6	28.1	**510.87**	4.5	28	**522.22**
Vitamin E (mg)	9.1	9.2	1.10	12.8	12.8	0.00	8.7	8.7	0.00
Minerals									
Calcium (mg)	1073.5	1077.8	0.40	1120.9	1125	0.37	1397.7	1401.6	0.28
Iodine (µg)	210.2	210.3	0.05	219.1	219.1	0.00	260.3	260.3	0.00

Iron (mg)	12.2	12.6	3.28	12.3	12.8	4.07	8.6	9	4.65
Magnesium (mg)	375.1	386.5	3.04	392.3	403.8	2.93	347	358.6	3.34
Phosphorus (mg)	1715.2	1817.5	5.96	1801.8	1907.3	5.86	1776.6	1886.9	6.21
Potassium (mg)	3676.3	4023.9	9.46	3907.9	4263.5	9.10	3845.6	4212.6	9.54
Selenium (µg)	75.2	89.7	**19.28**	81.4	96.5	**18.55**	64.8	80.7	**24.54**
Sodium (mg)	1404.9	1413.85	0.64	1501.9	1511.2	0.62	1178.5	1187.7	0.78
Zinc (mg)	13.8	13.9	0.72	14.1	14.2	0.71	11.9	12	0.84
**Ovo-lacto vegetarian diet**									
Macronutrients									
Energy (kJ)	7144.6	7222.58	1.09	8043.3	8121.03	0.97	6391.6	6469.85	1.22
Protein (g)	88.2	90.385	2.48	91.7	93.975	2.48	85.4	87.645	2.63
Carbohydrate (g)	186.3	187.68	0.74	211.9	213.33	0.67	162.3	163.71	0.87
Sugars (g)	70.8	70.93	0.18	94.3	94.4	0.11	83.1	83.21	0.13
Dietary fibre (g)	48.5	49.9625	3.02	50.5	51.9725	2.92	37.3	38.7625	3.92
Total fat (g)	57.8	58.1725	0.64	68.3	68.7025	0.59	52	52.3925	0.75
SFA (g)	14.2	14.2625	0.44	17.6	17.6725	0.41	15.6	15.6325	0.21
Vitamins									
Thiamin (mg)	2.1	2.17	3.33	2.1	2.2	4.76	1.5	1.56	4.00
Riboflavin (mg)	2.4	2.36	-1.67	2.5	2.49	-0.40	2.6	2.58	-0.77
Niacin eq.(mg)	43.8	47.54	8.54	46.8	50.62	8.16	40.5	44.44	9.73
Vitamin B6 (mg)	1.7	1.67	-1.76	2	2	0.00	1.5	1.51	0.67
Folate (DFE) (µg)	682.4	701.22	2.76	703.6	722.35	2.66	622.8	641.62	3.02
Vitamin B12 (µg)	3.4	3.39	-0.29	3.7	3.74	1.08	5.3	5.3	0.00
Vitamin A (RE) (µg)	1172.1	1174.42	0.20	1324.3	1325.82	0.11	1080.1	1080.97	0.08
Vitamin C (mg)	83.3	84.77	1.76	128.9	130.07	0.91	83.6	84.12	0.62
Vitamin D (µg)	1.5	24.9	**1560.00**	2.2	25.6	**1063.64**	2.1	25.6	**1119.05**
Vitamin E (mg)	11	11.05	0.45	13.4	13.39	-0.07	8.7	8.67	-0.34
Minerals									
Calcium (mg)	1041	1044.62	0.35	1195.9	1199.4	0.29	1295.2	1298.66	0.27
Iodine (µg)	173.4	173.43	0.02	198.5	198.49	-0.01	218.1	218.09	0.00
Iron (mg)	17.1	17.56	2.69	16.1	16.58	2.98	12.4	12.85	3.63
Magnesium (mg)	542.4	553.93	2.13	559	570.51	2.06	462.1	473.7	2.51
Phosphorus (mg)	1793	1901.5	6.05	1915.1	2025.27	5.75	1787.2	1899.68	6.29

Potassium (mg)	3520.7	3882.5	**10.28**	4246	4611.87	8.62	3598.7	3970.26	**10.32**
Selenium (µg)	58	73.6	**26.90**	65.4	81.26	**24.25**	47.7	64	**34.17**
Sodium (mg)	1187.6	1196.55	0.75	1341.9	1351.02	0.68	1071.3	1080.37	0.85
Zinc (mg)	12.3	12.39	0.73	12.7	12.73	0.24	11.1	11.2	0.90

Nutrients showing a percentage change of at least 10% are shown in bold.
DFE, dietary folate equivalents; eq, equivalents; FDN, foundation; UV-MR, UV-exposed mushrooms; RE, retinol equivalents; SFA, saturated fatty acids; %Δ, percentage change in modelled diet compared to original Foundation diet.

References

1. Byron A, Baghurst K, Cobiac L, Baghurst P, Magarey A. A Modelling System to Inform the Revision of the Australian Guide to Healthy Eating. Canberra, Australia: National Health and Medical Research Council; 2011.

2. Australian Bureau of Statistics. Vitamin D: Australian Bureau of Statistics; 2013 [Available from: https://www.abs.gov.au/articles/vitamin-d.

3. Food Standards Australia New Zealand. Australian Food Composition Database - Release 2.0: FSANZ; [Available from: https://www.foodstandards.gov.au/science/monitoringnutrients/afcd/Pages/foodsearch.aspx.

4. National Health and Medical Research Council. Eat for Health Educator Guide. 2013.
